# The Application of Membrane Separation Technology in the Pharmaceutical Industry

**DOI:** 10.3390/membranes14010024

**Published:** 2024-01-17

**Authors:** Ruirui Ma, Juan Li, Ping Zeng, Liang Duan, Jimin Dong, Yunxia Ma, Lingkong Yang

**Affiliations:** 1 State Key Laboratory of Environmental Criteria and Risk Assessment, Chinese Research Academy of Environmental Sciences, Beijing 100012, China; mrr_1208570479@163.com; 2 Institute of Water Ecology and Environment, Chinese Research Academy of Environmental Sciences, Beijing 100012, China; 3 Qilu Antibiotic Pharm, Jinan 250105, China; jimin.dong@qilu-pharma.com (J.D.); yunxia.ma@qilu-pharma.com (Y.M.); lingkong.yang@qilu-pharma.com (L.Y.)

**Keywords:** membrane technology application, pharmaceutical industry, separation and purification, wastewater resources recycling

## Abstract

With the advancement in membrane technology, membrane separation technology has been found increasingly widespread applications in the pharmaceutical industry. It is utilized in drug separation and purification, wastewater treatment, and the recycling of wastewater resources. This study summarizes the application history of membrane technology in the pharmaceutical industry, presents practical engineering examples of its applications, analyzes the various types of membrane technologies employed in the pharmaceutical sector, and finally, highlights the application cases of renowned international and Chinese membrane technology companies in the pharmaceutical field.

## 1. Introduction

Membrane technology has undergone a long, historical development in laboratory research and achieved its first major industrial application in the 1960s [1]. Membrane is a kind of material with a selective separation function, and can transfer one component and restrict others because of the special properties of the components [2]. The main membrane technologies include microfiltration, ultrafiltration, nanofiltration, reverse osmosis, pervaporation, electrodialysis, and membrane bioreactors [3]. Membrane separation technology has unique advantages such as a high efficiency, wide applicability, and minimal environmental impact [2]. With a rapid development over the past few decades, membrane separation technology has become one of the emerging technologies and has been used in numerous industrial sectors.

With the rapid development of membrane technology, the application of membrane separation technology in the pharmaceutical industry is becoming increasingly widespread. Researchers have found extensive applications in pharmaceutical processes such as drug purification, wastewater treatment, and wastewater resource utilization [4,5]. The scientific and reasonable applications of membrane separation technology in the pharmaceutical field can improve production efficiency, ensure the progressiveness of pharmaceutical processes, reduce pollution, and promote the pharmaceutical industry’s development towards a greener, environmentally friendly, and highly efficient direction.

This study presents a comprehensive review on the application of membrane separation technology in the pharmaceutical industry. The application history of membrane technology in the pharmaceutical industry is summarized, and the practical engineering cases are presented. The various types of membrane technologies employed in the pharmaceutical sector are analyzed. The application cases of renowned domestic and international membrane technology companies in the pharmaceutical field are introduced. Conclusively, existing challenges and prospects of membrane separation technology within the field of pharmaceutical industry are identified.

## 2. History of Membrane Technology Application in the Pharmaceutical Industry

The application of membrane technology in the pharmaceutical field originated with the preparation of pharmaceutical-grade water. In 1972, GOW from the UK used cellulose acetate reverse osmosis membranes to produce pyrogen-free water [6]. Following this, in 1974, the U.S. pharmaceutical company Upjohn employed Dupont-produced, aromatic polyamide hollow fiber, reverse osmosis membrane modules to manufacture injectable water, meeting regulatory standards for injection water [7]. The 19th edition of the U.S. Pharmacopeia in 1975 introduced membrane methods for injectable water production, challenging the traditional belief in the reliability of distillation alone [8]. In the 1980s, membrane technology gradually extended to the purification and production of pharmaceuticals. Millipore, in the early 1980s, applied a combination of membrane filtration techniques to separate and refine cephalosporin C from fermentation broth [9]. In 1983, an ultrafiltration system developed by Japan’s Asahi Chemical Industry was reported, which used hollow fiber membranes for drug purification [10]. Subsequently, membrane materials like hollow fiber, ultrafiltration, and reverse osmosis membranes were applied for the extraction of antibiotics, vitamins, and proteases [11,12,13,14].

**Figure 1:**
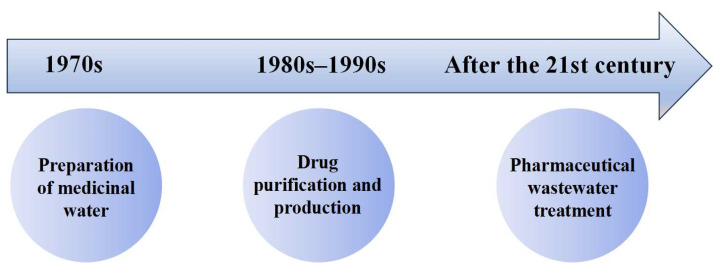
International application history of membrane technology in the pharmaceutical industry.

Since entering the 21st century, membrane separation technology has been gradually used in pharmaceutical wastewater treatment. A sewage treatment plant in Queensland used microfiltration and reverse osmosis technology to treat eleven kinds of drugs and two kinds of endocrine disruptors from different treatment categories. The results showed that the microfiltration and reverse osmosis systems can reduce the concentration of pollutants by an order of magnitude, and the overall removal efficiency of the circulating water is higher than 97% [15]. In addition, researchers proposed combining membrane separation technology with biological treatment units for pharmaceutical wastewater treatment [16,17,18]. After 2011, membrane technology in the pharmaceutical industry underwent a rapid development, with a surge in applications post-2016, notably focusing on the removal of pharmaceuticals, hormones, endocrine disruptors, and antibiotic resistance genes from wastewater [19]. An overview of the international application history of membrane technology in the pharmaceutical industry is depicted in Figure 1. In the 1970s, membrane technology was primarily employed for pharmaceutical-grade water preparation. Moving into the 1980s and 1990s, this transitioned towards drug purification and production. After entering the 21st century, there was a notable shift towards utilizing membrane technology for pharmaceutical wastewater treatment.

In China, the application of membrane technology in the pharmaceutical industry could be traced back to the 1960s. The Institute of Health Devices of the Academy of Military Medical Sciences (IHDAMMS) developed electrodialysis pure water equipment that uses multistage processes combined with pretreatment technologies of raw water to directly treat Tianjin tap water into injection water that meets pharmacopoeia standards [20]. In the late 1970s, with the advancement of membrane technology, the IHDAMMS began researching the use of reverse osmosis to produce pharmaceutical-grade water [21]. By the 1980s, membrane separation technology was also applied to the extraction of traditional Chinese medicines. In 1979, the pharmacy bureau of the PLA Air Force Beijing Hospital utilized membrane separation technology for the extraction of effective components from traditional Chinese medicines, demonstrating the efficacy of ultrafiltration in removing impurities while retaining the main constituents [22]. In 1981, they further utilized this method to prepare traditional Chinese medicine injections [23]. In the late 1990s, membrane technology found its application in antibiotic production. In 1989, Dalian Pharmaceutical Factory introduced Danish DDS sanitary reverse osmosis membrane equipment to concentrate the decolorization solution of streptomycin sulfate [9]. In 1994, the NFB series plate-type reverse osmosis device from the Penglai reverse osmosis equipment factory was successfully applied in streptomycin production at Jining Antibiotic Factory, demonstrating an improved quality, higher yield, and reduced energy and material consumption [24]. In 1999, Santar Membrane achieved a breakthrough in the crucial technology for cephalosporin production, developing a membrane separation-based process for 7-ACA production [25]. In 2000, Northeast Pharmaceutical General Factory utilized Ultra-Flo ultrafiltration membrane systems for the removal of impurities in a vitamin C fermentation broth, showing the potential of the membrane separation technology in shortening production processes, reducing costs, and increasing yields [26]. Subsequently, the application of membrane separation technology expanded to the production of other antibiotics like erythromycin and penicillin [27,28]. After entering the 21st century, with the rapid development of membrane technology, membrane separation techniques were further applied to the treatment of pharmaceutical wastewater. Since 2010, membrane bioreactor technology has been widely employed for pharmaceutical wastewater treatment, realizing multiple engineering applications as indicated in Table 1.

**Figure 2:**
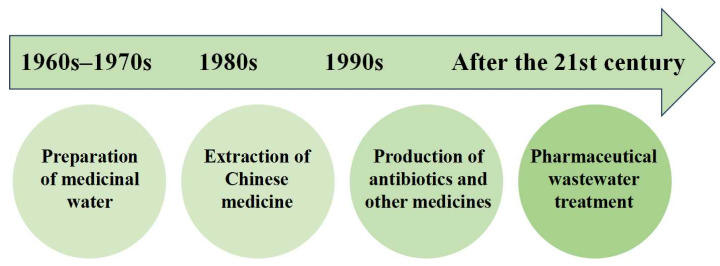
Chinese application history of membrane technology in the pharmaceutical industry.

The Chinese journey of membrane technology in the pharmaceutical industry, as illustrated in Figure 2, shows its evolution from the 1960s to 1970s primarily in pharmaceutical-grade water preparation to its gradual application in traditional Chinese medicine extraction in the 1980s, antibiotic production in the 1990s, and eventually, wastewater treatment in the 21st century.

## 3. Application of Membrane Technology in the Pharmaceutical Industry

The application of membrane technology in the pharmaceutical industry primarily encompasses microfiltration, ultrafiltration, nanofiltration, reverse osmosis, membrane bioreactors, electrodialysis, osmosis, osmotic vaporization, and combined processes. These membrane technologies are mainly used for pharmaceutical production, wastewater treatment, and wastewater product recovery, as shown in Table 2.

Microfiltration is the earliest membrane technology. The separation mechanism of microfiltration is sieving with a membrane pore size of 0.01–1 μm, which can allow macromolecular organic compounds and dissolved solids to pass through [2]. It is mainly used to intercept particles, bacteria, and pollutants from the liquid or gas phase to achieve the purposes of purification and bacteria removal [48]. In the pharmaceutical industry, microfiltration is mainly used for sterilization filtration, clarification of pharmaceutical solutions, removal of particles and viruses, purification of medical water, and pretreatment of ultrafiltration and reverse osmosis processes [49]. Among them, a microfiltration membrane with a pore size of 0.6–0.8 μm can be used for the removal of bacteria and the filtration of gases. A microfiltration membrane with a pore size of 0.45 μm is the most commonly used, often used for the purification of liquid material and water, and a microfiltration membrane with a pore size of 0.2 μm can be used for the sterilization filtration of liquid medicine [49].

Ultrafiltration is a kind of membrane filtration method with a molecular level membrane having a pore size of 10–100 nm. It uses the pressure difference on both sides of the membrane to selectively separate solutes with different molecular weights. In the pharmaceutical industry, ultrafiltration can not only be used to remove bacteria, viruses, and particles, but also pyrogens, hyphae, and proteins. It is often used for the fractionation and desalting concentration of macromolecular substances, the purification of small molecular substances, and the depyrogenation of pharmaceutical and biochemical preparations [50].

Nanofiltration is a membrane separation process between ultrafiltration and reverse osmosis, with a membrane pore size of 1–10 nm. The separation mechanism of nanofiltration is adsorption–diffusion. In the pharmaceutical industry, nanofiltration can be used for clarification, sterilization, filtration, protein removal, and the separation and purification of fermentation broths such as those for antibiotics, vitamins, amino acids, and enzymes [51]. It can also be used for the desalination and concentration of 6-APA, 7-ACA, 7-ADCA, and other semisynthetic antibiotics [49].

Reverse osmosis is also a membrane filtration process driven by pressure. The pore size of a reverse osmosis membrane is less than 1 nm, and its function is to intercept ionic substances that only pass through the solvent. The organic and inorganic molecules are separated from the feed solution through a solution diffusion process [52]. In the pharmaceutical industry, reverse osmosis is mainly used for drug concentration, purification and separation, desalination, preparation of water for preparation, water for injection, dialysis water, and sterile water [50].

Membrane bioreactor is a new wastewater treatment technology that combines membrane separation technology with biological treatment [53]. This technology adopts an immersion membrane component with a unique structure that is placed in an aeration tank. After aerobic aeration and biological treatment, the wastewater is pumped through a filter membrane and then pumped out [54]. In the pharmaceutical industry, membrane bioreactors are often combined with other technologies for the treatment of pharmaceutical wastewater [55].

**Table 2:** Application of different process types in the pharmaceutical industry.

Membrane Technology Types	Application Areas	Earliest Application Time	References
Microfiltration	Pharmaceutical production	1994	[56]
	Wastewater treatment	2005	[57]
	Wastewater product recovery	2015	[58]
Ultrafiltration	Pharmaceutical production	1983	[10]
	Wastewater treatment	2004	[59]
Nanofiltration	Pharmaceutical production	2003	[60]
	Wastewater treatment	1993	[61]
	Wastewater product recovery	2003	[62]
Reverse Osmosis	Pharmaceutical production	1984	[63]
	Wastewater treatment	2003	[64]
	Wastewater product recovery	2017	[65]
Membrane Bioreactor	Wastewater treatment	1995	[66]
	Pharmaceutical production	2008	[67]
Electrodialysis	Pharmaceutical production	2020	[68]
	Wastewater treatment	2019	[69]
	Wastewater product recovery	2013	[70]
Osmosis	Wastewater treatment	2011	[71]
	Wastewater product recovery	2015	[72]
Osmotic Vaporization	Wastewater treatment	2016	[73]
	Wastewater product recovery	2006	[74]
Combined Processes	Wastewater treatment	1987	[75]
	Pharmaceutical production	2004	[76]
	Reuse of reclaimed water	2011	[77]

## 4. Development of Membrane Technology and Its Application in the Pharmaceutical Industry by International Membrane Technology Enterprises

### 4.1. Pall Corporation (USA)

Pall Corporation was founded in New York in 1946. It specializes in the research of fluid purification and separation technologies and the production of filtration systems. Pall Corporation is the world leader in fluid filtration technology. Pall’s products are widely used in water treatment, and in biomedical, aerospace, petrochemical, food and beverage, metallurgical power, and microelectronics industries. Pall is the world’s largest filtration, separation, and purification company, involving the most extensive fields.

Pall Life Sciences comprises three divisions: biopharmaceutical, medical, and food and beverage. The biopharmaceutical department provides equipment and consumables for the entire process, from laboratory research and development to pilot testing and large-scale production. It is the only company in the world with a full line of pharmaceutical process fluid technologies. Pall first introduced the P grade (pharmaceutical grade) standard into the production of pharmaceutical filters, which has been widely adopted by the pharmaceutical industry. At the same time, Pall is the preferred filter supplier of GSK, Baxter, Frensenius, and other well-known pharmaceutical companies in the world.

### 4.2. Novasep (France)

Novasep is a company dedicated to the downstream separation and purification process solutions for the life science industry. Based on its core technologies such as the high-performance chromatography separation technology and crossflow membrane filtration technology, Novasep has developed a series of new production processes with low operating costs and environmental protection in the fields of medicine, food, bioengineering, dairy products, and starch deep processing. It has been used by thousands of companies in more than 50 countries around the world.

Novasep offers a wide range of services to the pharmaceutical industry. The company can provide competitive development and manufacturing solutions for the synthesis and purification of APIs, highly active APIs, and intermediates through a variety of flexible equipment, technical expertise, and advanced process capabilities. Novasep is a premium supplier in specific fields such as hazardous reactions, cytotoxic compounds, highly active pharmaceutical ingredients, low-temperature reactions, coupled reactions, etc. It can also carry out the production of various nonpatented pharmaceutical ingredients and intermediates such as nitroglycerin, paclitaxel, and omega-3.

### 4.3. Millipore (USA)

Millipore was founded in 1954 and mainly produces filter membrane and membrane filtration products. Milipore’s products can be divided into four major categories based on their application scope: laboratory pure water, life sciences, membrane technology, and bioengineering and pharmaceuticals. In the field of bioengineering and pharmaceuticals, Millipore can provide pharmaceutical companies with various specifications for filtration, ultrafiltration, reverse osmosis systems, chromatography systems, chromatography columns, and chromatography fillers for the clarification, separation, purification, and concentration of pharmaceutical and biological products and the removal of bacteria, pyrogens, and viruses from the final product. In July 2010, the German Merck Group successfully acquired the American company Millipore and established a new division: Merck Millipore. Merck Millipore is a new division of the Merck Group and is dedicated to the field of life science, with more than 40,000 kinds of products, basically covering all aspects of life science research, drug development, and production. Merck Millipore has thus become one of the three major suppliers of life science products in the world.

## 5. Development of Membrane Technology and Its Application in the Pharmaceutical Industry by Chinese Membrane Technology Enterprises 

### 5.1. Santar Membrane

Santar Membrane (Xiamen, China) is a pioneer in the field of membrane technology development and application in China. As early as the 1990s, Santar Membrane began to engage in the development of advanced membrane separation application technology in China and introduced foreign advanced membrane technology into China and carried out large-scale industrial applications. In 1998, Santar Membrane successfully developed a vitamin C production process based on membrane separation, realizing the dream of China’s leading position in vitamin C production technology. In 1999, Suntar Membrane broke through the major key technology of cephalosporin production, and successfully developed the production process of 7-ACA based on the membrane separation process. Santar Membrane is one of the first enterprises to promote the application of nanofiltration technology in the pharmaceutical industry in China and is also one of the first enterprises to apply ultrafiltration, nanofiltration, and continuous separation technologies to the production of vitamin C, penicillin, and 7-ACA in China. At present, Santar Membrane is widely used in the production of vitamins, antibiotics, traditional Chinese medicines, and biological products. Typical engineering cases for the application of Santar Membrane in the pharmaceutical industry are shown in Table 3.

### 5.2. Jiangsu Jiuwu High-Tech Co., Ltd.

Jiangsu Jiuwu High-tech Co., Ltd. (Nanjing, China) is the main pioneer of the ceramic film industry in China. Focusing on the development and application of membrane materials such as ceramic membranes and membrane separation technology, it is a well-known membrane industry enterprise with independent intellectual property rights. In the pharmaceutical field, Jiangsu Jiuwu High-tech Co., Ltd. has rich application experience in the separation and purification of antibiotics and the extraction of traditional Chinese medicines. In 2000, Jiangsu Jiuwu High-tech Co., Ltd. successfully applied the first set of threonine extractions in theindustry in China. In 2004, this company successfully applied the first set of ceramic membrane in the cephalosporin antibiotic extractions in China. Subsequently, Jiangsu Jiuwu High-tech Co., Ltd. successfully applied ceramic membranes to antibiotic production enterprises such as cephalosporins, erythromycin, and vancomycin, as well as to enzyme and pharmaceutical intermediate production enterprises. At the same time, this company has also successfully applied the ceramic membrane to the extraction of traditional Chinese medicines such as Rhodiola, Isatis indigotica, Salvia miltiorrhiza, and Pueraria lobata. The typical engineering cases of Jiangsu Jiuwu High-tech Co., Ltd. in the pharmaceutical industry are shown in Table 4.

### 5.3. Hangzhou Qiushi Membrane Technology Co., Ltd.

Hangzhou Qiushi Membrane Technology Co., Ltd. (Hangzhou, China) is one of the largest suppliers of membrane products in China, specializing in the research and development, production, sales, and technical services of all kinds of ultrafiltration membrane, and is one of the largest manufacturers of PVDF membrane and PP membrane in China. The applications of Hangzhou Qiushi Membrane Technology Co., Ltd. in the pharmaceutical industry mainly include the separation of fermentation liquid and the treatment of pharmaceutical wastewater. The typical engineering cases of this company’s applications in the pharmaceutical industry are shown in Table 5.

## 6. Conclusions and Prospect

Membrane technology provides a feasible alternative to conventional separations, purification, and wastewater treatment in pharmaceutical processing. It has been widely used in drug production, pharmaceutical wastewater treatment, and wastewater recycling. The high efficiency of membrane technology provides an alternative for pharmaceutical production. The economic feasibility and advancement of MBR technology provide a better option for the treatment of pharmaceutical wastewater over the traditional methods [4]. However, the application of membrane technology is still limited with the main drawback of membrane fouling [4]. With the continuous progress of membrane technology, it will inevitably become more advanced and perfect. The pharmaceutical industry should continuously introduce the most advanced membrane separation technology and develop its new applications, so as to maximize its advantages in the pharmaceutical field and promote the sustainable development of the pharmaceutical industry.

## Figures and Tables

**Table 1:** Engineering cases of membrane technology applications in the pharmaceutical industry.

Year	Treatment Process	Membrane Module	Scale (m^3^/d)	Wastewater Type	Influent Quality	Effluent Quality	Investment and Operating Cost Analysis	Ref.
2010	Anoxic + aerobic + MBR process	SMM-1520 PVDF membrane	2000	Fermented pharmaceutical wastewater	COD_Cr_ 18,000 mg/L, BOD_5_ 7200 mg/L	COD_Cr_ < 120 mg/L, BOD5 < 40 mg/L	-	[29]
2010	Contact oxidation + hydrolysis + MBR	PVDF hollow fiber membrane	385	Wastewater from chemical synthesis of cephalosporin antibiotics	pH 5.2–10.5, COD 2125–11,561 mg/L, BOD_5_ 421–3356 mg/L, TN 34.98–299.72 mg/L, NH_4_^+^-N 0.09–5.46 mg/L, NO_3_^−^-N 9.77–81.12 mg/L, TP 3.90–156.96 mg/L, TDS 567–7876 mg/L	COD 79–282 mg/L, BOD_5_ < 10 mg/L, TN 8.90–148.13 mg/L, NH_4_^+^-N 1.88–161.56 mg/L, NO_3_^−^-N 2.15–75.11 mg/L	-	[30]
2011	Coagulation precipitation + MBR	-	120	Pharmaceutical wastewater of Chinese patent medicines	pH 5–7, COD 3000–6000 mg/L, BOD_5_ 1500–2000 mg/L, SS 450 mg/L	pH 6–9, COD < 100 mg/L, BOD_5_ < 20 mg/L, SS < 20 mg/L	Total project investment: USD 294,000. Treatment cost: 0.25 USD/m^3^.	[31]
2013	Hydrolytic acidification + A/O + MBR process	Double-layered, large-area flat film	360	Integrated biomedical wastewater	pH 6–9, COD 400–600 mg/L, BOD_5_ 100–200 mg/L, SS 150–250 mg/L, NH_4_^+^-N 30–70 mg/L, TP 4–9 mg/L	pH 6–9, COD < 50 mg/L, BOD_5_ < 10 mg/L, SS< 5 mg/L, NH_4_^+^-N < 10 mg/L, TP < 0.5 mg/L	Total project investment: USD 187,880. Operating cost: 0.11 USD/m^3^.	[32]
2016	Comprehensive regulation + aerobic + MBR	SADF-2590 membrane	100	Pharmaceutical factory production equipment cleaning water, purified water preparation wastewater discharge, workshop floor washing water, waste gas treatment equipment cleaning water, and production workshop domestic sewage	pH 6–9, COD ≤ 1600 mg/L, BOD ≤ 480 mg/L, NH_4_^+^-N ≤ 30 mg/L, TN ≤ 40 mg/L, SS ≤ 100 mg/L, petroleum products ≤ 10 mg/L	pH 6–9, COD < 30 mg/L, BOD < 7 mg/L, NH_4_^+^-N < 5 mg/L, TN < 14 mg/L, SS < 9 mg/L, petroleum products < 1 mg/L	Total project investment: USD 840,000. Cost of wastewater treatment: 0.168 USD/m^3^. Cost of reclaimed water reuse: 0.21 USD/m^3^.	[33]
2016	Pretreatment + hydrolytic acidification + UASB + secondary A/O + MBR	-	800	Chemical synthetic pharmaceutical production wastewater	COD_Cr_ 35,955 mg/L, BOD_5_ 5000 mg/L, NH_4_^+^-N 1141 mg/L	COD_Cr_ 84 mg/L, BOD_5_ 16 mg/L, NH_4_^+^-N 7 mg/L	Total project investment: USD 3,206,000. Treatment cost: 7.86 USD/m^3^.	[34]
2016	Advanced oxidation + hydrolytic acidification + MBR + activated carbon filtration process	-	600	High concentration of pharmaceutical wastewater that is difficult to degrade	pH 6–9, COD 920 mg/L, BOD_5_ 360 mg/L, SS 200 mg/L, NH_4_^+^-N 15 mg/L, TN 25 mg/L, TP 1.4 mg/L	pH 6–9, COD 30 mg/L, BOD_5_ 6 mg/L, SS10 mg/L, NH_4_^+^-N 1.5 (2.5) mg/L, TN 10 mg/L, TP 0.3 mg/L	Operational cost is 0.532 USD/m^3^.	[35]
2017	Flat film MBR process	Flat membrane	500	Biological pharmaceutical wastewater	COD 400 mg/L, BOD_5_ 200 mg/L, SS 50 mg/L, TN 62 mg/L, NH_4_^+^-N 32 mg/L, TP 1.2 mg/L	COD ≤ 80 mg/L, BOD_5_ ≤ 10 mg/L, SS ≤ 1 mg/L, TN ≤ 30 mg/L, NH_4_^+^-N ≤ 10 mg/L, TP ≤ 0.5 mg/L	Electricity cost for operation: 0.14 USD/m^3^. Membrane cleaning chemical cost: 0.002 USD/m^3^. Operation and maintenance cost: 0.15 USD/m^3^.	[36]
2017	Anaerobic + A/O + sand filter + carbon filter + ultrafiltration + reverse osmosis + triple-effect evaporation	-	150	Biological pharmaceutical wastewater	pH 5–6, COD 7632 mg/L, NH_4_^+^-N 106 mg/L, TP 17 mg/L, SS 157 mg/L	pH 6–9, COD 34 mg/L, NH_4_^+^-N 1.3 mg/L, TP 0.12 mg/L, SS 2.6 mg/L	The operating cost is 5.51–6.48 USD/m^3^.	[37]
2018	Hydrolytic acidification + efficient anaerobic reactor + biochemistry + MBR	-	4000	Chemical synthesis of raw material production wastewater	COD 6000 mg/L	COD 300 mg/L	Operational cost is 22.68 USD/m^3^.	[38]
2019	Air floating + compound oxygen + MBR process	Hollow fiber membrane	100	Biological pharmaceutical wastewater	pH 4–9, COD_Cr_ 500–8000 mg/L, BOD_5_ 200–3000 mg/L, NH_4_^+^-N 10–500 mg/L, TP 1–60 mg/L, SS 300–2000 mg/L	pH 6–9, COD_Cr_ 86 mg/L, BOD_5_ 3.9 mg/L, NH_4_^+^-N 9.8 mg/L, TP 0.46 mg/L, SS ≤ 70 mg/L	Total project investment: USD 157,920. Operational cost: 1.52 USD/m^3^.	[39]
2019	Pretreatment + ABR + A/O + MBR	-	240	High concentration, biochemical pharmaceutical wastewater	pH 5–6, COD 3000 mg/L, BOD_5_ 300 mg/L, NH_4_^+^-N 180 mg/L, SS 1000 mg/L	pH 6–9, COD ≤ 200 mg/L, BOD^5^ ≤ 150 mg/L, NH_4_^+^-N ≤ 20 mg/L, SS ≤ 100 mg/L	-	[40]
2019	AA/O + MBR	-	100	Pharmaceutical wastewater	pH 6–9, COD 2820–5910 mg/L, SS 210–620 mg/L, petroleum products 52–80 mg/L	pH 6–9, COD 2820–5910 mg/L, SS 210–620 mg/L, petroleum products 52–80 mg/L	Total investment: USD 107,800. Operational costs: 0.37 USD/m^3^.	[41]
2020	Biochemical treatment + ultrafiltration + reverse osmosis + ion exchange resin	-	9600	High-salt pharmaceutical wastewater	pH 6–9, COD 294–816 mg/L, BOD 120–300 mg/L, NH_4_^+^-N 71–150 mg/L, TP 10–20 mg/L, SS 120–1100 mg/L	-	Total investment: USD 7 million. Treated effluent meets cooling water standards (operating costs: 0.63 USD/m^3^) and secondary desalination water standards (operating costs: 1.26 USD/m^3^).	[42]
2020	Electrodialysis + steam recompression technology	-	96	High salt and high ammonia nitrogen pharmaceutical wastewater	pH 5–6, COD 15,000–25,000 mg/L, 3%–5% NH_4_Cl	-	The total investment: USD 1.4 million. Annual operating cost: USD 0.28 million.	[43]
2020	Biochemical treatment + membrane treatment (ultrafiltration + reverse osmosis) + ion exchange resin	SMT600-P50 ultrafiltration membrane; W30XFR-400/34i and SW30XLE-400iB reverse osmosis membrane	400	Biological pharmaceutical wastewater	pH 7.0–9.0, COD 60–80 mg/L, BOD_5_ 10–20 mg/L, NH_4_^+^-N 15–20 mg/L, TP 1–2 mg/L, Cl^−^ 200–350 mg/L, TDS 4500–6000 mg/L	pH 6.5–9.0, COD ≤ 60 mg/L, BOD_5_ ≤ 10 mg/L, NH_4_^+^-N ≤ 5 mg/L, TP ≤ 1 mg/L, CL-X ≤ 250 mg/L, TDS ≤ 1000 mg/L, EC ≤ 0.15 μS/cm, SiO_2_ ≤ 10 μg/L	The total project investment: USD 5.32 million. Treated effluent meets cooling water standards (operating costs: 0.63 USD/m^3^) and secondary desalination water standards (operating costs: 1.26 USD/m^3^).	[44]
2020	Multi-effect clarifier + ultrafiltration + recovery system	-	7500	Biochemical secondary sedimentation tank effluent	pH 6.5–8, COD_Cr_ 200–500 mg/L, BOD_5_ 20–200 mg/L, SS350 mg/L, TDS 3000–6000 mg/L, hardness 500 mg/L, TN 50–70 mg/L	-	The treatment cost is 0.55 USD/m^3^.	[45]
2021	A combined process of coagulation sedimentation + AAO + MBR + ozone contact oxidation + high-efficiency air floatation + activated carbon adsorption + contact disinfection	PVDF hollow fiber membrane	2000	Biopharmaceutical wastewater	COD 150 mg/L, BOD_5_ 60 mg/L, TN 20 mg/L, NH_3_-N 15 mg/L, TP 6.0 mg/L, SS 250 mg/L	COD 20 mg/L, BOD_5_ 8 mg/L, TN 1.0 mg/L, NH_3_-N 0.5 mg/L, TP 0.1 mg/L, SS 5 mg/L	Operational cost is 1.39 USD/m^3^.	[46]
2023	Biotreatment + MBR	-	8000	High salinity and high concentration organic wastewater in pharmaceutical industry	COD_Cr_ 8700–9300 mg/L, SS 3600–4250 mg/L	COD_Cr_ 270–290 mg/L, SS 170–190 mg/L	The treatment cost is 3.45 RMB/m^3^.	[47]

**Table 3:** Typical engineering cases of Santar Membrane in the pharmaceutical industry.

Project Name	Membrane Technology	Scale (m^3^/d)	Application Type	Source
Erythromycin nanofiltration concentration project of a pharmaceutical company	Nanofiltration	3840	Desalination, dewatering, and concentration process of erythromycin filtrate	[78]
Amino acid treatment project of a Xinjiang company	Ceramic membrane filtration + nanofiltration concentration	100	Amino acid production	
Erythromycin nanofiltration concentration project of a biotechnology company	Nanofiltration	5700	Erythromycin concentration production	
Membrane treatment technology of erythromycin of a Ningxia pharmaceutical company	Ceramic membrane and nanofiltration membrane technology	-	Ceramic membrane technology is applied to clarify impurities in red mold antibiotic fermentation broth, and nanofiltration membrane technology is applied to desalination and concentration of red mold antibiotic filtrate.	

**Table 4:** Typical engineering cases of Jiangsu Jiuwu High-tech Co., Ltd. in the pharmaceutical industry.

Project Name	Scale (m^3^/d)	Application Type	Source
Jiaozuo Jiankangyuan Bioproducts Co., Ltd.	320	Fermentation broth separation	[79]
Huabei Pharmaceutical Hebei Huamin Pharmaceutical Co., Ltd.	300	Fermentation broth separation	
Lunan New Era Biotechnology Co., Ltd.	520	Fermentation broth separation	
Lizhu Group Ningxia Fuxing Pharmaceutical Co., Ltd.	400	Fermentation broth separation	
Yili Chuanning Biotechnology Co., Ltd.	400	Fermentation broth separation	
Huabei Pharmaceutical Hebei Huamin Pharmaceutical Co., Ltd.	300	Continuous filtration of fermentation broth by ceramic membrane	

**Table 5:** Typical engineering cases of Hangzhou Qiushi Membrane Technology Co., Ltd. in the pharmaceutical industry.

Project Name	Membrane Technology	Scale (m^3^/d)	Application Type	Source
Shandong Pulodebang Pharmaceutical Co., Ltd.	MBR	1500	Pharmaceutical wastewater treatment	[80]
Hebei Jianmin MBR System	MBR	12,000	Pharmaceutical wastewater treatment	
Hebei Jianmin Starch MBR Project	MBR	6000	Pharmaceutical wastewater treatment	
Shanghai Pharmaceutical Co., Ltd.	Ultrafiltration + reverse osmosis	760	Separation of sugar fermentation broth	

## Data Availability

No new data were created or analyzed in this study. Data sharing is not applicable to this article.

## References

[1] Fane A.G., Wang R., Jia Y., Wang L.K., Chen J.P., Hung Y.T., Shammas N.K. (2011). Membrane Technology: Past, Present and Future. Membrane and Desalination Technologies.

[2] Wang C., Wang Y., Qin H., Lin H., Chhuon K. (2020). Application of Microfiltration membrane Technology in Water treatment. IOP Conf. Ser. Earth Environ. Sci..

[3] Wee S.-L., Tye C.-T., Bhatia S. (2008). Membrane separation process—Pervaporation through zeolite membrane. Sep. Purif. Technol..

[4] Ankush, Mandal M.K., Sharma M., Khushboo S., Pandey S., Dubey K.K., Bui X.T., Chiemchaisri C., Fujioka T., Varjani S. (2019). Membrane Technologies for the Treatment of Pharmaceutical Industry Wastewater. Water and Wastewater Treatment Technologies. Energy, Environment and Sustainability.

[5] Liu H.B., Li B., Guo L.W., Pan L.M., Zhu H.X., Tang Z.S., Xing W.H., Cai Y.Y., Duan J.A., Wang M. (2022). Current and Future Use of Membrane Technology in the Traditional Chinese Medicine Industry. Sep. Purif. Rev..

[6] (1976). Production of Solutions. British Patent.

[7] Juberg D.L., Pauli W.A., Artiss D.H. (1977). Application of reverse osmosis for the generation of water for injection. Bull. Parenter. Drug Assoc..

[8] United States Pharmacopeial Convention (1975). The United States Pharmacopoeia XIX.

[9] Hao C., Huang X. (2004). Membrane separation technology and its application in pharmaceutical production. Chem. Pharm. Eng..

[10] Warashina T., Hashino Y., Kobayashi T. (1983). Development of Hollow-Fiber Type Ultrafiltration System. Research and Development in Japan Awarded the Okochi Memorial Prize.

[11] Toussaint G., Ding L.H., Jaffrin M.Y., Hassairi I., Nonus M. (2000). Recovery of α-Agarase Enzyme from Fermentation Broths by Membrane Crossflow Filtration. Sep. Sci. Technol..

[12] Manrong J., Pingli L., Qinghui S., Shichang W. (1995). Composite hollow fiber membrane and its application in concentrating vitamine B12. Water Treat..

[13] Chaufer B., Rollin M., Grangeon A., Dulieu-Barton J.M. (1992). Tetracycline Removal or Concentration with an Inorganic Ultrafiltration Membrane Modified by Quaternized Polyvinylimidazole Coating. Key Eng. Mater..

[14] Stremovskii L.L., Navashin S.M. (1984). Purification and separation of benzylpenicillin and phenoxymethylpenicillin by ultrafiltration through a semipermeable membrane. Dokl. Chem. Technol..

[15] Al-Rifai J., Khabbaz H., Schäfer A.I. (2011). Removal of pharmaceuticals and endocrine disrupting compounds in a water recycling process using reverse osmosis systems. Sep. Purif. Technol..

[16] Kimura K., Hara H., Watanabe Y. (2007). Elimination of selected acidic pharmaceuticals from municipal wastewater by an activated sludge system and membrane bioreactors. Environ. Sci. Technol..

[17] Quintana J.B., Weiss S., Reemtsma T. (2005). Pathways and metabolites of microbial degradation of selected acidic pharmaceutical and their occurrence in municipal wastewater treated by a membrane bioreactor. Water Res..

[18] Clara M., Strenn B., Ausserleitner M., Kreuzinger N. (2004). Comparison of the behaviour of selected micropollutants in a membrane bioreactor and a conventional wastewater treatment plant. Water Sci. Technol. A J. Int. Assoc. Water Pollut. Res..

[19] Ren S., Boo C., Guo N., Wang S., Elimelech M., Wang Y. (2018). Photocatalytic Reactive Ultrafiltration Membrane for Removal of Antibiotic Resistant Bacteria and Antibiotic Resistance Genes from Wastewater Effluent. Environ. Sci. Technol..

[20] Gong C. (1999). Review and prospect of membrane production of medicinal water. Membr. Sci. Technol..

[21] Gong C., Li M., Dai F., Sun X., Sun S., Chen W. (1982). Water for injection is prepared by reverse osmosis. Mil. Med. Sci..

[22] The Pharmacy Bureau of the PLA Air Force Beijing Hospital (1979). Chinese herbal medicine injection was prepared by ultrafiltration technology. Chin. Tradit. Herb. Drugs.

[23] The Pharmacy Bureau of the PLA Air Force Beijing Hospital (1981). Experimental study on preparation of compound Chinese medicine injection by ultrafiltration method. Chin. Tradit. Herb. Drugs.

[24] Zhang Z., Wang S., Jiang Z., Jing Y., Lin Y., Chu Q. (1994). Application of plate reverse osmosis device in streptomycin production process. Technol. Water Treat..

[25] Li C., Fang F., He X., Xia H., Lan W. (2001). Application of Ultrafiltration for Purification of Cephalosporin C. J. Fujian Med. Univ..

[26] Zhang L., Gao Y., Li J. (2000). Application of ultrafiltration in improvement of the vitamin C production process. Membr. Sci. Technol..

[27] Wang X., Zhang C., Zhao J. (2000). Separation mechanism of nanofiltration membranes and its applications in food and pharmaceutical industries. Membr. Sci. Technol..

[28] Li X., Luan B., Han G. (1996). Application of ultrafiltration in the extraction of antibiotics. Technol. Water Treat..

[29] Huang W., Zhou R., Liao Z. (2010). Reconstruction of pharmaceutical fermentation wastewater treatment project. Ind. Water Treat..

[30] Zheng W., Chen L., Yao C., Li Y., Li W. (2010). Treatment of Wastewater in Chemical Synthesis of Cephalosporin by Contact Oxidation-Hydrolysis-MBR Process. Environ. Prot. Chem. Ind..

[31] Su Y., Lin F. (2011). Treatment of pharmaceutical wastewater by coagulation precipitation—MBR process. Water Wastewater Eng..

[32] Huang H., Xu H., Zhou B., Yang W., Huang J., Cheng Z. (2013). Hydrolysis Acidification-A/O-MBR Process for Treatment of Biopharmaceutical Wastewater. China Water Wastewater.

[33] Xu G., Liu W., Zhong L., Tu K., Luo X. (2016). Project Case of Intermediate Water Recovery for Membrane Bioreactor Treatment Process of One Pharmaceutical Wastewater. Mod. Chem. Res..

[34] Feng J. (2016). Engineering Example of Pharmaceutical Wastewater Treatment. Guangdong Chem. Ind..

[35] Ma Y., Liu X., Feng H., Zhang J., Zhai Y., Wang L. (2016). Pharmaceutical wastewater treatment process design and operational example. China Water Wastewater.

[36] Zhao Z. (2017). The Application of the Flat Sheet Membrane MBR Process in the Modification of Pharmaceutical Wastewater Treatment Plant. Guangdong Chem. Ind..

[37] Chen H., Ren X., Zhang W., Liu X. (2017). Engineering Project of the Treatment and Reuse of Biological Pharmaceutical Wastewater. Technol. Water Treat..

[38] Xu J., Liu F., Yang H. (2018). Engineering Example of A Pharmaceutical Wastewater Treatment. Guangdong Chem. Ind..

[39] Xu F. (2019). Treatment of biopharmaceutical wastewater by air floating-compound oxygen-MBR process. Ind. Water Wastewater.

[40] Zhang H., Zhang F. (2019). Pharmaceutical wastewater treatment process design example. Energy Conserv..

[41] Fu J. (2019). Pharmaceutical wastewater treatment upgrading project. Plant Maint. Eng..

[42] Han Z. (2020). Case study on advanced treatment of high salt pharmaceutical wastewater and reuse of boiler make-up water in power plant. Electr. Power Technol. Environ. Prot..

[43] Wang M., Yu K., Liu G., Zhang J. (2020). Engineering example of treatment of high salinity and high ammonia wastewater by electrodialysis + MVR combined technology. Coal Chem. Ind..

[44] Han Z. (2020). A project example of advanced treatment and reuse of biopharmaceutical wastewater. Ind. Water Wastewater.

[45] Zhang Z. (2020). Engineering example of pharmaceutical wastewater reuse and zero discharge. Resour. Econ. Environ. Prot..

[46] Shen X.-L. (2021). An example of project design of biopharmaceutical wastewater advanced treatment. Ind. Water Wastewater.

[47] Zeng G.L., Li J. (2023). Application case of MBR process in high salinity and high concentration organic wastewater treatment in pharmaceutical industry. Water Purif. Technol..

[48] Baker R.W. (2012). Membrane Technology and Applications.

[49] Hao C., Huang X. (2004). Application of membrane technology in pharmaceutical production. J. Filtr. Sep..

[50] Xue G., Hu X., Chen X., Zheng X. (2009). Applications of Membrane Separation Technology in the Production of Medicine and Medical Treatment. Chem. Ind. Eng..

[51] Mallakpour S., Azadi E. (2022). Nanofiltration membranes for food and pharmaceutical industries. Emerg. Mater..

[52] Peters T. (2010). Membrane Technology for Water Treatment. Chem. Eng. Technol..

[53] Gu Y.L., Huang J.H., Zeng G.M., Shi L.X., Shi Y.H., Yi K.X. (2018). Fate of pharmaceuticals during membrane bioreactor treatment: Status and perspectives. Bioresour. Technol..

[54] Yu X., Sun Y. (2019). Application of membrane bioreactor technology in the transformation of biopharmaceutical wastewater treatment system. Chin. J. Biol..

[55] Nasrollahi N., Vatanpour V., Khataee A. (2022). Removal of antibiotics from wastewaters by membrane technology: Limitations, successes, and future improvements. Sci. Total Environ..

[56] Goldner H. (1994). Pharmaceutical Processes Benefit From New Filtration Systems. RD Mag..

[57] Doll T.E., Frimmel F.H. (2005). Cross-flow microfiltration with periodical back-washing for photocatalytic degradation of pharmaceutical and diagnostic residues-evaluation of the long-term stability of the photocatalytic activity of TiO_2_. Water Res..

[58] Samaia M., Chikhi M., Bouzerara F. (2015). Elimination of Penicillin V by Membrane Process. Chem. Eng. Trans..

[59] Li S.Z., Li X.Y., Wang D.Z. (2004). Crystallization of oxytetracycline from fermentation waste liquor: Influence of biopolymer impurities. J. Colloid Interface Sci..

[60] Zhang W., He G.H., Gao P., Chen G.H. (2003). Development and characterization of composite nanofiltration membranes and their application in concentration of antibiotics. Sep. Purif. Technol..

[61] Treffry-Goatley K., Gilron J. (1993). The application of nanofiltration membranes to the treatment of industrial effluent and process streams. Filtr. Sep..

[62] Zhu A., Zhu W.P., Wu Z., Jing Y.F. (2003). Recovery of clindamycin from fermentation wastewater with nanofiltration membranes. Water Res..

[63] McBain D. (1984). Reverse-Osmosis Offers Many Advantages. Water Serv..

[64] Reinhard M., Montgomery-Brown J., Louie J.S., Gross B. (2003). From effluent to new water: Performance evaluation and quality assurance. Chimia.

[65] Hsieh D.S., Lindrud M., Lu X.J., Zordan C., Tang L.Y., Davies M. (2017). A Process for Active Pharmaceutical Ingredient Recovery from Tablets Using Green Engineering Technology. Org. Process Res. Dev..

[66] Benitez J., Rodriguez A., Malaver R. (1995). Stabilization and dewatering of wastewater using hollow fiber membranes. Water Res..

[67] Ong A.L., Kamaruddin A.H., Bhatia S., Aboul-Enein H.Y. (2008). Enantioseparation of (R,S)-ketoprofen using Candida antarctica lipase B in an enzymatic membrane reactor. J. Sep. Sci..

[68] Gao W.T., Chen Q., Du M.G., Zhang W.M., Cao C.Y., Song W.G. (2020). Enabling an atom-economic production of chiral amino alcohols by electrodialysis with bipolar membranes. Green Chem..

[69] Arola K., Ward A., Mänttäri M., Kallioinen M., Batstone D. (2019). Transport of pharmaceuticals during electrodialysis treatment of wastewater. Water Res..

[70] Ravikumar Y.V.L., Sridhar S., Satyanarayana S.V. (2013). Development of an electrodialysis-distillation integrated process for separation of hazardous sodium azide to recover valuable DMSO solvent from pharmaceutical effluent. Sep. Purif. Technol..

[71] Hancock N.T., Xu P., Heil D.M., Bellona C., Cath T.Y. (2011). Comprehensive Bench- and Pilot-Scale Investigation of Trace Organic Compounds Rejection by Forward Osmosis. Environ. Sci. Technol..

[72] Pan S.F., Zhu M.P., Chen J.P., Yuan Z.H., Zhong L.B., Zheng Y.M. (2015). Separation of tetracycline from wastewater using forward osmosis process with thin film composite membrane—Implications for antibiotics recovery. Sep. Purif. Technol..

[73] Woldemariam D., Kullab A., Fortkamp U., Magner J., Royen H., Martin A. (2016). Membrane distillation pilot plant trials with pharmaceutical residues and energy demand analysis. Chem. Eng. J..

[74] Kreis P., Górak A. (2006). Process analysis of hybrid separation processes: Combination of distillation and pervaporation. Chem. Eng. Res. Des..

[75] Cai B.X., Lang K.M., Liu Y.R., Chen Y.M. (1987). The refinement and concentration of agricultural antibiotic A in aqueous solution using membrane processes. Desalination.

[76] Li S.Z., Li X.Y., Wang D.M. (2004). Membrane (RO-UF) filtration for antibiotic wastewater treatment and recovery of antibiotics. Sep. Purif. Technol..

[77] Joss A., Baenninger C., Foa P., Koepke S., Krauss M., McArdell C.S., Rottermann K., Wei Y., Zapata A., Siegrist H. (2011). Water reuse: >90% water yield in MBR/RO through concentrate recycling and CO_2_ addition as scaling control. Water Res..

[78] https://www.suntar.com/application/cases/5.

[79] http://www.jiuwu.com/gc.html.

[80] http://www.creflux.net/?mod=case&classid=1.

